# A qualitative study of patient experiences: socioeconomic barriers to radiotherapy access in Gauteng, South Africa

**DOI:** 10.3332/ecancer.2025.2043

**Published:** 2025-11-20

**Authors:** Portia N Ramashia, Pauline B Nkosi, Thokozani P Mbonane

**Affiliations:** 1 Department of Environmental Health, Faculty of Health Sciences, University of Johannesburg, Johannesburg 2000, South Africa; 2 Department of Medical Imaging and Radiation Sciences, Faculty of Health Sciences, University of Johannesburg, Johannesburg 2000, South Africa; 3 Faculty of Health Sciences, Durban University of Technology, Durban 4000, South Africa

**Keywords:** cancer care, Gauteng Province, healthcare, health inequities, radiotherapy access, South Africa, treatment delays, socio-economic challenges, Sub-Saharan Africa sustainable development goal

## Abstract

Cancer poses a significant and growing health challenge in Sub-Saharan Africa, contributing to increasing mortality rates. Access to radiotherapy, a critical component of effective cancer treatment, remains severely limited, thereby hindering progress towards achieving Sustainable Development Goal (SDG) 3, which aims to reduce premature mortality from non-communicable diseases, including cancer. This qualitative study explores the multifaceted challenges faced by patients in accessing radiotherapy services within Gauteng Province, South Africa, emphasising the urgent need for a comprehensive framework to improve access. Employing a cross-sectional qualitative design, in-depth interviews were conducted with 25 cancer patients undergoing radiotherapy to investigate the socio-economic and demographic barriers impacting their ability to access treatment. The study revealed significant barriers, including the limited availability of radiotherapy facilities, inefficient referral processes and substantial financial burdens. These challenges were often exacerbated by patients’ socio-economic status and geographic location, thereby highlighting disparities in access to care. Data from the other phases indicates long waiting times for consultations and treatment, and there are only two public radiotherapy facilities in the province, primarily concentrated in urban areas, with limited equipment and trained personnel to meet the growing demand. The findings underscore the pressing need for targeted interventions to enhance radiotherapy access in Gauteng Province, such as strategic investments in infrastructure, streamlining referral pathways and addressing financial constraints through targeted support programs. Addressing these challenges requires a multi-pronged approach involving government, healthcare providers and community organisations. Comprehensive strategies and increased investment are essential to improve access to radiotherapy in Sub-Saharan Africa, which is crucial for achieving SDG 3 and reducing cancer-related mortality. Without scaling up access, many lives will be unnecessarily lost to this treatable disease

## Background

Cancer is a growing global health concern, posing a significant threat to health and well-being worldwide [1, 2]. Access to radiotherapy, a crucial component of comprehensive cancer care, remains profoundly inequitable, particularly in low- and middle-income countries (LMICs) like those in sub-Saharan Africa [3, 4]. This disparity in access directly undermines progress towards achieving Sustainable Development Goal (SDG) 3, which aims to ensure healthy lives and promote well-being for all ages. Specifically, target 3.4 focuses on reducing premature mortality from non-communicable diseases, including cancer, by one-third by 2030 [5]. In South Africa, and particularly in Gauteng Province, these global challenges are acutely felt, where this research reveals critical challenges to accessing radiotherapy services for cancer patients. These challenges include the scarcity of facilities, long travel distances, financial burdens and delays in diagnosis and treatment initiation [6, 7].

Gauteng Province, while being the wealthiest province in South Africa, faces a significant cancer burden [8]. According to the National Cancer Registry, the province accounts for approximately 25% of all cancer cases in South Africa, with breast, prostate, cervical and lung cancers being the most prevalent. The crude incidence rate in 2017 was 87.5 per 100,000, while the mortality rate was 43.8 per 100,000 [9]. In 2022, among females, the most frequent types of cancer diagnosed were breast cancer, cervical cancer and colorectal cancer [10]. These statistics highlight the urgent need for accessible and timely cancer care services. However, access to radiotherapy services remains a critical challenge in Gauteng. Data from the other phases indicate long waiting times for consultations and treatment, and there are only two public radiotherapy facilities in the province, primarily concentrated in urban areas, with limited equipment and trained personnel to meet the growing demand. This scarcity translates to prolonged waiting times and limited access for patients, particularly those in underserved communities, hindering efforts to reduce cancer-related mortality [6, 7].

This study investigates patient experiences with accessing radiotherapy services in Gauteng. It aims to understand the barriers that affect patients’ ability to receive timely and appropriate treatment. By exploring the patient’s perspectives, this research seeks to inform interventions and policies aimed at improving access to radiotherapy and reducing inequities in cancer care. The insights from this study, combined with findings from the other phases of the project, specifically Improving Access to Radiotherapy Services in Gauteng: Quantitative Analysis of Key Time Intervals from Diagnosis to Treatment and Improving Access to Radiotherapy: Exploring Structural Quality Indicators for Radiotherapy in Gauteng Province, South Africa, provide a foundation for an actionable framework.

This paper represents the third phase of a larger, multi-phased project aiming to develop a comprehensive framework to improve radiotherapy access in Gauteng Province, with earlier phases focusing on a systematic review of barriers to radiotherapy access in Sub-Saharan Africa, quantitative analysis of key time intervals from diagnosis to treatment, and exploration of structural quality indicators. This study explores the challenges cancer patients face in accessing radiotherapy services in Gauteng Province through Penchansky and Thomas’s [11] 5 A’s framework. This framework suggests that access to healthcare is multifaceted, influenced by five key dimensions: Availability, which refers to the existence of adequate services and re-sources, including radiotherapy facilities, equipment and trained personnel, to meet the needs of the population; Accessibility, which encompasses the geographic and structural capacity to reach services, including factors like distance to facilities, transportation options and ease of navigating the healthcare system; Accommodation, which refers to the fit between how services are organised and delivered and the needs and preferences of patients, including appointment scheduling, waiting times, communication and cultural sensitivity; Affordability, which refers to the financial capacity of patients to access services, including the direct costs of treatment and indirect costs like transportation, accommodation and lost income; and acceptability, which refers to the compatibility between patient and provider attitudes, values and beliefs, encompassing factors such as trust, communication, cultural sensitivity and perceptions of the quality of care [11]. Therefore, this study seeks to answer the following research question: What are the lived experiences of cancer patients in Gauteng Province, South Africa, regarding the socio-economic and demographic challenges they face in accessing radiotherapy services? By exploring these five dimensions of access, this study aims to provide a comprehensive understanding of the barriers faced by cancer patients in accessing radiotherapy services in Gauteng Province.

By exploring patient experiences and identifying key socio-economic and demo-graphic challenges related to availability, accessibility, accommodation, affordability and acceptability of radiotherapy services, this study aims to provide evidence-based recommendations for enhancing cancer care equity and advancing progress towards SDG 3 in sub-Saharan Africa.

## Methods

### Study approach and design

This study employed a descriptive qualitative design to deeply explore the multifaceted challenges faced by cancer patients accessing radiotherapy services in Gauteng Province [12]. While this approach provides a rich, in-depth understanding of complex phenomena, its descriptive nature and single-time point data collection limit the ability to establish causality or examine changes over time. A descriptive qualitative design was chosen because we believed that focusing on the experiences, perspectives and individual narratives was the right way to understand complex social phenomena [12, 13]. While a longitudinal design would have provided valuable insights into changes in access over time, it was not feasible due to the limited duration of the research project and resource constraints. The data collected at a single time point offers a valuable ‘snapshot’ of the challenges patients face at this moment [12]. This baseline data is crucial for informing future research and interventions aimed at improving radiotherapy access in Gauteng.

Efforts were made to minimise potential biases through careful interview design, triangulation of data sources and rigorous data analysis techniques [14]. However, it is important to acknowledge the potential for recall bias, as participants were asked to recall past experiences. Future research should focus on longitudinal studies to examine changes in access over time, as well as quantitative studies to quantify the impact of specific interventions on improving radiotherapy access in Gauteng.

### Setting and population

This qualitative study explores the socio-economic and demographic factors influencing access to radiotherapy services in Gauteng Province, South Africa. Gauteng Province, the most populous and economically active province, contributes approximately 35% to South Africa’s GDP. Gauteng Province has a population exceeding 12 million, making it the most populous province in South Africa [8]. The study focuses on radiotherapy patients at two public radiotherapy facilities in Gauteng Province. Participants were recruited from both treatment and follow-up settings over 2 months. The specific demographics of the study participants, such as age, gender, race and socio-economic status, will be further described in the Results section.

### Sampling

Due to the exploratory nature of this study and the significant challenges associated with recruiting participants in resource-constrained settings, a convenience sampling method was employed to recruit participants presenting for radiotherapy treatment or follow-up at the two participating radiotherapy centres. This approach aligns with the principles of qualitative descriptive research, which seeks to provide a comprehensive summary of events and their meaning for participants [15]. Qualitative description is a suitable goal when a straight description of a phenomenon is desired or information is sought to develop and refine questionnaires or interventions [13]. The flexible approach of this design allows some elements of other qualitative approaches to be employed [15]. As a result, this sampling technique ‘allows the researcher to complete interviews or get responses cost-effectively [16].’ The sample size was determined by data saturation, rather than a predetermined number of participants. Data saturation was initially achieved after 25 interviews, as no new themes or insights emerged from subsequent interviews. To confirm that data saturation had been achieved and to ensure the robustness of our findings, we conducted two additional interviews at each of the participating radiotherapy centres. These additional interviews did not yield any new themes or insights, further supporting the conclusion that data saturation had been reached [17].

It is important to acknowledge the potential for selection bias introduced by convenience sampling, as participants who were more accessible or willing to participate may not be representative of the broader population of radiotherapy patients [15]. To mitigate this bias, we collected demographic data to assess the representativeness of the sample, comparing our findings with existing literature and being transparent about the limitations of our sampling method. Despite these limitations, the in-depth qualitative data obtained through this sampling approach provides valuable insights into the lived experiences of patients accessing radiotherapy services in Gauteng Province, contributing to a deeper understanding of the challenges they face.

### Data collection and research measures

This study was approved by the Faculty of Health Sciences Research Ethics Committee (REC) at the University of Johannesburg (REC-2509-2023). To ensure ethical conduct, several measures were implemented to protect participant privacy and confidentiality. All patients’ information was anonymised, replacing identifying details with unique codes. Data were stored securely on password-protected computers, and access was restricted to the research team.

Potential participants from the two radiotherapy centres in Gauteng Province were invited through paper-based invitations distributed by the researcher. Specifically, the researcher provided the invitations to patients during their radiotherapy or follow up visit in the waiting area with the assistance of the radiotherapists and the nurses. Interested individuals indicated their interest by indicating they would like to participate. Face-to-face interviews were conducted with cancer patients on treatment and follow-up at two public radiotherapy facilities in Gauteng Province. Data collection occurred over 2 months, with participants recruited from treatment and follow-up settings. As such, this study provides a cross-sectional view of patient experiences, capturing a range of perspectives at a single point in time. Future research could benefit from longitudinal data collection to understand how access barriers evolve over the course of treatment and survivorship.

Participants were eligible for inclusion if they met the following criteria: diagnosed with one of the five most prevalent cancers in the region (breast, head and neck, prostate, gastrointestinal and cervical), on treatment or follow-up at two public radiotherapy facilities in Gauteng Province, aged 18 years or older and willing to voluntarily participate in the study and had signed informed consent. The selection of these five cancer types was based on their prevalence in Gauteng Province and South Africa. The reason for focusing on these common cancers was aimed at capturing a range of experiences relevant to a significant proportion of radiotherapy patients in the region, providing valuable insights for service improvement efforts [18].

The interview guide was developed based on Penchansky and Thomas’s [11] 5 A’s framework, which provides a comprehensive approach to understanding the various dimensions of access to healthcare services, namely: Availability, Accessibility, Affordability, Acceptability and Accommodation. The interview questions were structured to explore each of these dimensions. For example, questions related to travel time to the facility are related to Accessibility**.** The development process involved a review of relevant literature and was further informed by our systematic review of barriers to radiotherapy access in Sub-Saharan Africa [19] and consultations with radiation oncology stakeholders and decisions made by the research team regarding the specific questions to be included. The interview guide is included as Appendix A [11]. Interviewers underwent training to ensure consistent application of the interview guide and to minimise potential bias in data collection.

All patients’ information was anonymised for privacy. An information letter outlining the study’s purpose was shared with participants, and informed consent was obtained before the interviews, which lasted for 20–30 minutes. With the participant’s consent, interviews were audio-recorded, transcribed verbatim and coded for thematic analysis. Additionally, field notes were taken to capture contextual details and non-verbal cues. This approach aligns with recommendations for qualitative research, emphasising the depth and richness of data over a large sample size [17, 20].

### Data management and analysis

**Figure 1.**
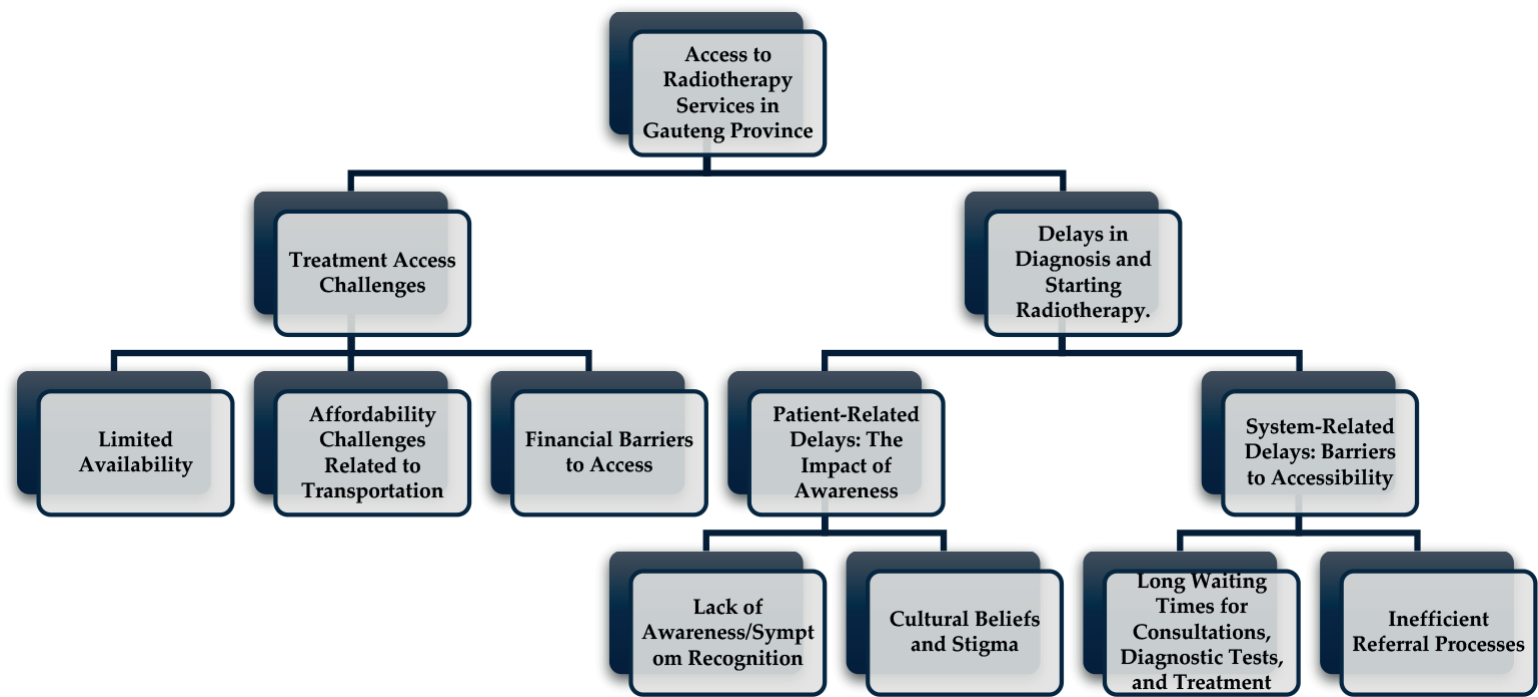
Structure of themes and subthemes.

A total of 45 codes were identified in the analysis of the interview transcripts. Atlas.ti software (version 24) was used to manage and organise the interview transcripts, facilitate the coding process and support the identification of themes [21]. The interview transcripts were independently coded by two researchers. Inter-coder reliability was assessed, with discrepancies in coding resolved through discussion and consensus to ensure consistent application of codes. A six-phase inductive thematic analysis, as outlined by Clarke and Braun [22], was employed to analyse the transcribed interview data. This approach allowed themes to emerge directly from the data, reflecting participants’ lived experiences regarding access to radiotherapy services. Initial codes were grouped into potential themes based on shared meaning and conceptual relationships. These themes were then reviewed against the coded data to ensure they accurately reflected the participants’ experiences. The themes were refined through an iterative process of discussion and revision among the research team. To further illustrate this process, Table 1 provides a snapshot of our analysis, demonstrating the progression from raw data to initial codes and, ultimately, to broader thematic categories. Thematic analysis, as outlined by Clarke and Braun [22], was chosen for its flexibility and ability to identify patterns across a diverse set of qualitative data. This approach allowed us to explore the multifaceted experiences of cancer patients regarding access to radiotherapy services, providing rich insights into their challenges and needs. To visually represent the thematic structure, Figure 1 maps the relationships between the main themes and sub-themes identified in the analysis. While thematic analysis provides valuable insights into participants’ experiences, it is important to acknowledge that the interpretation of themes is inherently subjective. To mitigate this limitation, we employed a rigorous and transparent coding and theme development process, involving multiple researchers and systematic validation procedures.

To ensure rigor and trustworthiness, an independent data transcriber and coder was employed. This individual was responsible for transcribing the interview data verbatim and then coding all the transcripts. The independent coder reached complete agreement with the primary researcher. Therefore, there were no discrepancies in coding that required resolution. Furthermore, member checking was employed to validate the findings with participants. The Consolidated Criteria for Reporting Qualitative Research checklist guided the reporting of the study’s findings [23].

### Ethical considerations

This study was approved by the Faculty of Health Sciences REC at the University of Johannesburg (REC-2509-2023). The ethics committee reviewed the study protocol, informed consent procedures, interview questions and data storage and security measures. All participants provided written informed consent before their involvement in the study. Participants were provided with a detailed information sheet outlining the study’s purpose, procedures, potential risks and benefits and their right to withdraw at any time. They had the opportunity to ask questions and were given sufficient time to consider their participation. To protect participants’ confidentiality, all data were anonymised, and participants were assigned pseudonyms in any reports or publications. Data were stored securely on password-protected computers accessible only to the research team. The researchers engaged in reflexivity throughout the study, acknowledging their own perspectives and biases and how these might have influenced the research process. This study was conducted in accordance with the principles of the Declaration of Helsinki.

## Results

The experiences of patients undergoing radiotherapy were explored through semi-structured interviews conducted at two public oncology centres in Gauteng Province. Twenty-five patients were interviewed: 22 who were currently receiving treatment and 3 who were attending follow-up appointments. Participants ranged in age from 19 to 75 years, with a majority being female (84%). The most prevalent cancer type among participants was cervical cancer, reflecting its significant burden in this population. Most participants resided in townships and faced considerable travel distances, exceeding 30 km, to access the radiotherapy facilities.

Regarding socioeconomic and educational backgrounds, the participant group exhibited diversity. While 16% of participants held a degree, the majority had completed high school (48%) or some secondary education (24%). A smaller proportion had completed only primary school education (12%). A significant portion of participants were unemployed and reliant on pensions (40%), while 28% were employed in low-wage jobs. These factors highlight the potential for socioeconomic disparities to influence access to and experiences with radiotherapy. A detailed breakdown of the study population’s characteristics, including cancer types, socioeconomic status and other relevant demographics, is presented in Table 2.

This study explores the challenges faced by cancer patients in accessing radiotherapy services in Gauteng Province, South Africa, through the lens of Penchansky and Thomas’s [11] 5A’s framework of access. This widely recognised framework posits that healthcare access is influenced by five interconnected dimensions: **Availability** (the existence of adequate services and resources), **Accessibility** (the geographic proximity and ease of reaching services, as well as structural factors), **Accommodation** (the extent to which service organisation aligns with patient needs and preferences), **Affordability** (the financial capacity of patients to utilise services) and **Acceptability** (the compatibility of socio-cultural factors, attitudes and values between patients and providers).

Table 3 provides a concise overview of the key challenges identified in this study, categorised according to the 5A’s framework. The subsequent sections delve into these challenges in detail, providing specific examples and patient narratives to illustrate the impact of each dimension on access to radiotherapy in Gauteng.

The study’s key findings are organised around two major themes: Treatment access challenges and delays in diagnosis and starting radiotherapy.

### Treatment access challenges: availability and affordability

Access to radiotherapy is crucial for cancer care, but patients in resource-limited settings face significant challenges. This theme delves into the significant challenges cancer patients encounter in Gauteng Province seeking radiotherapy services. These challenges are not merely individual hardships but represent systemic issues within the healthcare landscape, often compounded by socio-economic disparities. This section will explore two crucial subthemes: the limited availability of radiotherapy facilities and patients’ affordability challenges. These obstacles, when combined, create a formidable barrier to accessing life-saving treatment, underscoring the urgent need for targeted interventions to improve access and equity in cancer care within Gauteng Province.

#### Limited availability and accessibility of radiotherapy facilities

The availability of radiotherapy facilities in Gauteng Province is a critical factor influencing access to this essential cancer treatment. International guidelines recommend one linear accelerator per 250,000 people; however, Gauteng faces challenges in meeting this target, particularly within the public sector [7]. While Gauteng Province is the economic hub of South Africa, the distribution of radiotherapy centres is not uniform, creating geographic disparities in access. The public sector facilities must cater to a large population, leading to strain. This limited availability often necessitates long travel distances for patients, particularly those residing in the townships. As shown in Table 2, participants resided as far as 114 km away from the nearest facility. The average distance travelled by participants in this study was 40 km to reach the nearest radiotherapy facility, with an average travel time of 2 hours. A significant number of patients rely on public transport, which is sometimes unreliable.

This scarcity often necessitates long patient travel distances or moving away from home for the duration of the treatment, creating financial and logistical burdens. The following excerpts from participants, support this:

*‘I take two taxis each way. I have to leave my house at 5 am so I can be one of the patients treated in the morning and avoid getting back home late at night.’ (C Participant 1, a 35-year-old female with Head & Neck cancer, and travels 40 km to the facility).*

This highlights the significant time commitment and logistical planning required for patients to access treatment.

*‘The journey is so long and tiring. By the time I get to the hospital, I am already exhausted before the treatment even begins.’ (S PT 3, a 54-year-old female with cervical cancer, travels 65 km).*

This illustrates the physical toll that long-distance travel takes on patients, potentially impacting their ability to tolerate treatment.

*Bara is actually the nearest hospital to us, and it is an academic hospital, so why don’t we have an oncology unit there? (C Participant 11, a 69-year-old male with prostate cancer, matriculated education, who travels 55km).*

This raises concerns about the strategic placement of radiotherapy facilities and the potential for decentralisation to improve access.

#### Affordability challenges related to transportation

The financial burden of accessing radiotherapy is substantial, further compounding the affordability challenge. 60% of participants reported transportation costs exceeding 120 rands per day. This represents a significant portion of the daily income for many low-income individuals in Gauteng. For instance, C Participant 5 is a domestic worker who travels 19 km to the facility – the taxi fares eat a large portion of her income. This forces patients to make difficult choices, often sacrificing necessities to afford treatment. When asked about their travel arrangements, participants indicated their challenges with transportation costs, which affected their ability to afford proper meals and medical appointments. They expressed frustration over the expenses due to having to attend their radiotherapy appointments consistently.

*‘Sometimes the taxi fare is more than I recieve. I have to choose between eating and getting to my treatment.’ (C Participant 9, a 72-year-old female with cervical cancer and a pensioner who travels 45km).*

This highlights the impossible choices faced by elderly patients with limited incomes.

*‘Mmmh, at home, I cannot eat properly because of this transport issue. I will not lie to you*’. *(C Participant 6, a 68-year-old female with breast cancer and a pensioner, who travels 14.2 km).*

This demonstrates the direct impact of transportation costs on basic nutrition.

When asked how long it took them to attend an appointment where a doctor referred them, participant 8 expressed that it took them a month due to the financial challenges of covering transport costs:

*‘It took me a month. Because I did not have money to go to Joburg’ (C Participant 8, a 50-year-old female with cervical cancer who travels 45 km).*

This illustrates how transport costs can create significant delays in accessing necessary medical care.

These quotes highlight the significant financial strain placed on patients due to transportation costs, directly impacting the affordability of treatment. The financial burden associated with travel can lead to treatment delays and interruptions, potentially compromising treatment outcomes.

#### Financial barriers to access

A significant proportion of patients in Gauteng Province face substantial financial barriers to accessing radiotherapy, with many earning low wages or being unemployed. Most of these patients rely on the already strained public healthcare system, specifically the two radiotherapy centres in Gauteng [7]. While the results highlight the significant financial challenges faced by cancer patients in Gauteng, several limitations warrant consideration. While the study provides valuable insights into the socio-economic challenges faced by cancer patients seeking radiotherapy in Gauteng Province, it is important to acknowledge certain limitations. The sample size of 25 patients, with the most common cancer type being cervical cancer, may limit the generalisability of our findings to broader cancer patient populations, particularly concerning specific cancer types such as breast cancer. Although the number of participants with breast cancer was not specifically quantified, the study focused on common socio-economic factors affecting access to treatment. However, the financial and logistical challenges described by participants were consistent across different cancer types, suggesting that certain barriers, such as transportation costs and loss of income, are common across diagnoses. Future research should aim to include larger, more diverse samples with specific attention to individual cancer types to allow for more detailed analysis.

Treatment often necessitates taking leave from work, with most participants relying on unpaid leave due to the inability to work during treatment. The loss of income due to treatment is particularly devastating for households reliant on a single income or those supporting multiple dependents.

*‘No, I used my leave days…you know how private operates if you have finished your sick leave and your other leave days, there is no payment.’ (S Participant 7, a 46-year-old female with cervical cancer working as a clerk).*

This highlights the precarious situation of those in the private sector with limited or no paid leave.

Financial strain was exacerbated by job insecurity and the inability to work during treatment. One participant explained how using all available leave days resulted in lost income due to the private sector’s lack of paid leave provisions. Others, reliant on small businesses, faced further hardship as treatment prevented them from working.

*‘There is nothing because I am here…’ (S Participant 10, a 58-year-old female with cervical cancer and primary school education, working as self-employed).*

This illustrates the complete loss of income experienced by some patients.

‘*It is no longer coming in. My husband’s things were not going well around January; they were only picking up now, so it was difficult because I was the one helping at home. You see, my husband is self-employed. What helped me is that NSFAS helped my child with school…’ (S Participant 7, a 46-year-old female with cervical cancer working as a clerk).*

This highlights how the loss of income impacts whole families.

*‘It takes time sometimes to process my document because I am a temporary teacher. So, it is very hard for me to fund my transport from where I stay to here. It is very hard, but I have to do that.’ (S Participant 11, a 42-year-old female with breast cancer working as a temporary teacher).*

This demonstrates the challenges faced by temporary workers.

Even with public healthcare covering treatment costs, expenses like transport to multiple appointments remained a significant burden. Some resorted to borrowing from family and friends, creating financial dependence.

*We have not been paid, and even when that money comes, you must notice that it is not spent by me alone. Children are not working, grandchildren are not working, and there is no work. When it comes, I take and add there. I buy gas, I buy electricity, right? I bought maize meal, and we will use this money at home. We cannot even do anything about it (C Participant 9, a 72-year-old female with cervical cancer and a pensioner who travels 45km).*

This quote illustrates how these expenses impact not only the patient but the entire extended family.

*‘I had to borrow money from my family just to pay for the taxi to the hospital. The treatment itself is free, but I cannot afford the travel costs.’ (C Participant 12, a 45-year-old female with cervical cancer and a general worker).*

This underscores the burden of transport costs.

These accounts underscore the significant financial barriers faced by cancer patients in Gauteng Province seeking radiotherapy. The costs associated with treatment, travel and lost income create immense hardship and can compromise treatment adherence and outcomes. Addressing these financial barriers is crucial for ensuring equitable access to life-saving cancer care.

### Delays in diagnosis and starting radiotherapy: the awareness gap

Timely diagnosis and prompt initiation of radiotherapy are critical for effective cancer treatment and improved patient outcomes. However, various factors can contribute to delays in this process, potentially jeopardising treatment success and increasing patient anxiety. This theme examines the multifaceted delays experienced by cancer patients in Gauteng Province, categorised into two key subthemes: Patient-Related Delays focusing on the Awareness aspect of access and System-Related Delays. Understanding these distinct yet interconnected sources of delay is crucial for developing targeted strategies to expedite the cancer care pathway and improve patient experiences.

#### Patient-related delays: the impact of awareness

Delays originating from the patient’s side often stem from a complex interplay of personal, social and cultural factors. These delays, while seemingly individual choices, can be heavily influenced by systemic inequalities and limited access to information and resources. This subtheme explores two key contributing factors:

### Lack of awareness/symptom recognition

Many participants reported a lack of awareness about cancer symptoms, leading to delays in seeking medical advice. This lack of awareness manifests in several ways:

Misinterpretation of Symptoms: Individuals may misinterpret cancer symptoms as signs of less serious illnesses, leading them to self-treat or delay seeking professional medical attention.

Normalisation of Symptoms: Individuals may normalise or downplay their symptoms, attributing them to age, stress or other factors. The downplay can be particularly true for vague or intermittent symptoms.

Limited Access to Information: Limited access to reliable health information, particularly in underserved communities, can hinder early symptom recognition and prompt help-seeking.

This lack of awareness and education about cancer management was identified as a problem. The lack of awareness would often result in participants not treating the symptoms with a sense of urgency, thus causing a delay in seeking medical attention. Participants would often dismiss the symptoms for other illnesses up until the situation became dire. The lack of awareness and education is seen in the following quotes:

*(laughs) However, I think I did not really pay that much attention. I did not look into it that much until the day I collapsed (S Participant 4, a 19-year-old male).*

*‘I did not know anything about cancer. I did not even know what the symptoms were.’ (C Participant 1, a 35-year-old female with Head & Neck cancer).*

*‘I thought it was just irregular menses. I did not think it could be anything serious.’ (S Participant 7, a 46-year-old female with cervical cancer working as a clerk).*

#### Cultural beliefs and stigma

Cultural beliefs and stigma surrounding cancer influenced how individuals interpreted and responded to symptoms significantly. In many communities, traditional healers play a significant role in healthcare, often offering alternative treatments for a variety of illnesses. However, reliance on traditional healing can sometimes delay access to conventional medical care, potentially impacting treatment outcomes. Participants shared that they would receive advice or non-medical treatment intervention from friends and family members, which would cause a delay in receiving treatment. For example, one participant explained:

*‘…so, after going to the clinic twice, my aunt told me I must go to a traditional healer because what I have is because I slept with someone’s husband’. (S Participant 6, a 26-year-old female with cervical cancer)*

Another participant indicated that they use traditional medicine as an alternative while waiting for a call back from the radiotherapy department.

*‘You know, as a black person, I was told to go consult, and others were telling me to take this ….I went back home to KZN, where I was at the traditional healer for 3 weeks because my husband suggested it since the hospital is not helping me.’ (C Participant 12, a 45-year-old female with cervical cancer).*

#### System-related delays: barriers to accessibility

Beyond individual patient factors, systemic inefficiencies and resource limitations within the healthcare system create significant barriers to accessing radiotherapy services. These delays, which directly impact accessibility, exacerbate existing healthcare disparities, disproportionately affecting vulnerable populations. This subtheme examines two key areas that highlight these accessibility challenges:

### Long waiting times for consultations, diagnostic tests and treatment

One of the most significant accessibility challenges identified by participants was the extensive waiting period to receive consultations, diagnostic tests and initiate radiotherapy treatment. These delays, often beginning from the moment patients first seek medical advice after noticing symptoms, can have serious consequences, potentially leading to later-stage diagnoses and poorer treatment outcomes. Participants reported substantial waiting times, with one individual waiting 6 months for a biopsy result and another enduring an 8-hour wait for a brief consultation. These delays are primarily attributed to resource limitations within the healthcare system, including critical shortages of healthcare professionals and limited access to essential diagnostic equipment such as imaging machines. This restricted access to timely diagnostic services creates a significant barrier to care. The following quotes illustrate the impact of these delays:

*‘I waited for months to see a specialist. By the time I started treatment, the cancer had already changed stage. It was on stage 2B, and when I started the treatment, it was stage 3’. (S Participant 7, a 46-year-old female with cervical cancer working as a clerk).*

The average diagnostic delay was 6 months, with some patients waiting up to 1 year. There was a further 2–3-month wait for some patients after their treatment planning computed tomography (CT) simulation scan before they commenced radiotherapy.

*‘The hospital here was in 2018. They made the appointment, but the appointment was only for September 2019’.(C Participant 11, a 69-year-old male with prostate cancer).*

### Inefficient referral processes

The referral process, intended to streamline access to specialised care, often becomes frustrating and confusing, further hindering accessibility. Patients often seek initial medical advice from their local clinics, resulting in their transfer to tertiary hospitals where treatment is offered. However, the referral process to tertiary healthcare or radiotherapy centres depends on how local clinic/hospital healthcare professionals perceive the urgency of the treatment. As a result, some cases are treated as a priority, resulting in a quicker referral, while others are not. This inconsistency creates an uneven playing field, where access to timely care depends on subjective assessments and potentially arbitrary prioritisation.

Furthermore, referrals to other hospitals are sometimes necessary for further examination by specialists, adding another layer of complexity to the already challenging process. This fragmented and inconsistent referral system significantly hinders accessible and timely radiotherapy services. One participant recounted their experience:

*‘The doctor told me to go to Helen Joseph, and then Helen Joseph said no, the situation I was in was not related to them. It is related to Rahima Moosa’. (C Participant 8, a 50-year-old female with cervical cancer and works as cleaner*)

Issues with scheduling and communication further complicate the radiation treatment process, highlighting the challenges participants and healthcare providers face in managing high patient volumes. Some participants felt confused, concerned and hurt during their hospital stay due to a lack of communication from the medical staff about their condition and treatment plan. This lack of communication is seen in Participant 5’s response below, who became emotional because of not receiving clarity regarding their cancer diagnosis.

*‘(Sobbing) when I got in, I found about 5 doctors who were waiting for me, and they said my Pap smear results did not look good. It has whatever H1 what what what. That means…hai! They did not even tell me what it meant. They just requested to examine me. They came with all their tools, and everyone examined what they wanted to examine’. (S Participant 5, a 43-year-old female with cervical cancer).*

*‘This experience further added to the weight of having to deal with the cancer symptoms. Again, clear communication and understanding between doctors and patients are necessary to avoid misunderstandings and ensure proper care. This transparency and effective communication from healthcare professionals could help patients navigate through the treatment. They must learn to tell someone that it is like this and that. I saw we saw what. What do we find about that person? That is why we did so’. (C Participant 9, a 72-year-old female with cervical cancer and a pensioner who travels 45km).*

*‘Yes, I feel like if they had addressed the issue when it was smaller, I would not have gotten to where I was. I feel like the traditional methods may have added things that were not meant to be there, the way I see it’. (C Participant 12, a 45-year-old female with cervical cancer).*

## Discussion

This research has revealed critical challenges to accessing radiotherapy services for cancer patients in Gauteng Province, South Africa. The scarcity of facilities, resulting in patients traveling long distances and incurring substantial financial burdens, coupled with delays in diagnosis and treatment initiation, remain key impediments to care [24, 25]. These challenges directly undermine efforts to achieve SDG 3, particularly Target 3.4, which aims to reduce premature mortality from non-communicable diseases, including cancer, by one-third by 2030 [5]. While this study does not offer a direct comparative analysis, the identified barriers resonate with existing literature on access to cancer care in other LMICs. Even if the barriers are not entirely novel, this study provides valuable context-specific insights into how these challenges manifest within the unique healthcare landscape of Gauteng Province. Furthermore, the identified barriers likely exacerbate existing health inequities, disproportionately affecting vulnerable populations. For example, lower socioeconomic status can significantly impact access, as individuals may struggle to afford transportation, out-of-pocket expenses and time off work. The intersectionality of these factors creates significant barriers, potentially leading to delayed diagnoses and poorer treatment outcomes for marginalised communities [19].

Our findings highlight the interconnectedness of availability, accessibility, affordability, accommodation and acceptability—the 5 A’s—in shaping patient experiences and ultimately impacting treatment outcomes. The limited availability of radiotherapy facilities resulted in substantial travel burdens, often necessitating costly and time-consuming journeys for patients. These financial burdens further compounded affordability challenges, creating significant barriers for patients, particularly those from lower socio-economic backgrounds. These financial and logistical obstacles are not unique to Gauteng Province but reflect broader access challenges across Sub-Saharan Africa [25]. Datta, Samiei and Bodis [26] projected significant shortages of radiotherapy infrastructure and human resources in LMICs, highlighting the global nature of this challenge. Similarly, Thompson *et al* [27] highlighted the impact of distance and travel time on access to radiotherapy, a concept often referred to as the ‘tyranny of distance. The situation in Ibadan, Nigeria, as described by Anakwenze *et al* [28], likely presents similar infrastructural challenges, given the broader context of radiotherapy access in Nigeria described by Akinwande *et al* [29]. The scarcity of resources, coupled with inefficient referral processes, contributed to substantial delays in diagnosis and treatment initiation, further emphasising the urgent need for systemic interventions. Furthermore, the lack of clear communication and support throughout the cancer care journey underscored the inadequacy of the system’s accommodation of patient needs. These findings resonate with existing literature documenting access barriers to radiotherapy in resource-constrained settings [30–33].

Regarding the feasibility and availability of free or subsidised accommodation and food for patients, this qualitative study vividly illustrates the profound need for such support. Our findings consistently highlight the substantial financial burdens faced by patients due to indirect costs such as transportation, accommodation and lost income. Patients often make difficult choices, often sacrificing necessities to afford treatment, including food [27]. Our recommendations explicitly call for targeted financial support programs for vulnerable patients, including subsidies for transportation and accommodation. While this paper does not assess the current availability or feasibility of such programs, it provides compelling qualitative evidence from patient experiences, establishing the critical importance and urgency of developing and implementing these interventions in future policy and programmatic work.

The public health implications of these findings are substantial. Limited access to radiotherapy perpetuates health inequities, disproportionately affecting vulnerable populations who already face significant barriers to healthcare. Delayed diagnosis and treatment resulting from access challenges can lead to poorer outcomes and increased mortality, exacerbating the cancer burden in South Africa [24]. Addressing these inequities requires a multifaceted approach. Investing in infrastructure development, particularly by increasing the number of radiotherapy facilities, is crucial. Balogun *et al* [25] discuss the need for equipment and upgrading existing machinery, which is a point highly relevant to resource-constrained settings. The findings of this echo systemic challenges highlighted by Donkor *et al* [34] particularly the challenges in coordinating care between different levels of the healthcare system and ensuring adequate resources for referral processes in LMICs [30]. Therefore, optimising referral pathways to streamline the process and reduce delays is essential. Further, innovative care models, such as mobile health, could enhance accessibility, particularly for patients in remote areas [24, 35].

The lack of clear communication leaves patients feeling lost and uninformed. Yap *et al* [36] emphasise the importance of clear communication and emotional support for patients navigating the complexities of cancer care, particularly in resource-constrained settings [36–39]. The lack of clear communication underscores the need for patient-centred care that addresses not only physical but also psychosocial needs. Furthermore, implementing financial assistance programs to alleviate the cost burden of treatment, travel and accommodation would make radiotherapy more affordable for vulnerable patients. Bhatia *et al* [39] mention a recent survey that can provide data for needs assessment and help inform future research; therefore, it can inform policy decisions on resource allocation.

Finally, fostering collaboration between conventional medical practitioners and traditional healers, coupled with community-based awareness campaigns to address cultural beliefs and stigma surrounding cancer, can enhance the acceptability of radiotherapy and encourage timely help-seeking. LaVigne *et al* [40] provide further context for this aspect within LMICs, which is applicable to the context of Sub-Saharan Africa [40–42].

This study represents a phase of a larger project focused on developing a comprehensive framework to improve radiotherapy access in Gauteng Province. Other phases of this project have examined structural quality indicators of radiotherapy services, including healthcare professional views [7] and analysed key time intervals from diagnosis to treatment [6]. The qualitative findings of this study, which explored patient experiences with radiotherapy access, are corroborated by findings from concurrent quantitative and mixed-methods phases of this research. For instance, the structural quality indicators of radiotherapy services in Gauteng employed a mixed-methods approach, analysing quantitative data from radiotherapy facilities and gathering qualitative insights through interviews with healthcare professionals. The study found that there are only two public radiotherapy facilities in Gauteng, compared to 18 private facilities. On average, public facilities operate with four linear accelerators and one brachytherapy unit, while private facilities, on average, have 1.27 linear accelerators and 0.27 brachytherapy units. All facilities reported having at least one CT simulator and magnetic resonance imaging machines were more prevalent in public facilities. Our analysis in that paper indicates a potential shortage, with Gauteng having approximately 2.09 linear accelerators per 1,000 patients (or 1.39 if only operational ones are counted) compared to the recommendation of 2.2 per 1,000 patients. This data corroborates our patients’ experiences regarding the limited availability of facilities, which often necessitates long travel distances and contributes to long waiting times [7]. Furthermore, the patient-reported delays are substantiated by the quantitative data from another phase of this research, Improving Access to Radiotherapy Services in Gauteng: Quantitative Analysis of Key Time Intervals from Diagnosis to Treatment, which provides insights into patient volumes within the public sector facilities. That study analysed 800 patient files (400 from each of the two public facilities) from January to December 2023, noting that ‘each facility treating approximately 4,000 patients annually’. This indicates a significant annual patient volume, which, when spread across the available machines and limited staff, underscores the pressure on the public healthcare system discussed in the current qualitative phase of the research. The study found that there was an average wait times exceeding 8 months for a first consultation with a radiation oncologist after confirmed biopsy diagnosis and 13 months to CT simulation for radiotherapy treatment planning [6]. While this paper does not provide a direct numerical estimation of required equipment or personnel, it provides crucial qualitative data on the impact of current shortages on patient experiences, particularly in terms of long waiting times for consultations, diagnostic tests and treatment. In addition, providing the foundational evidence upon which more precise estimations of required equipment and personnel can be developed in subsequent studies or by policymakers.

However, the limited scope of this study necessitates acknowledging its limitations. Focusing solely on Gauteng Province restricts the generalisability of our findings to the broader South African context, let alone Sub-Saharan Africa. Furthermore, the relatively small sample size warrants caution in generalising these findings. Future research should focus on multi-centre studies with larger, more diverse samples to assess the impact of specific interventions, such as financial assistance programs, on improving access and outcomes. Further studies should also explore facilitators of care and positive experiences to provide a more balanced picture of the patient’s journey. Finally, quantitative methods could be employed to measure the prevalence of these barriers and their impact on patient outcomes, as well as further investigating the role of socioeconomic factors and intersectionality in shaping patient experiences.

## Conclusion

This research confirms that significant challenges persist in accessing radiotherapy services in Gauteng Province, primarily due to facility scarcity, financial constraints and delays in treatment initiation. Our study uniquely illuminates the impact of these barriers on patient experiences, revealing the significant financial burdens and emotional distress caused by treatment delays. To address these issues, we recommend prioritising the streamlining of referral pathways through the implementation of electronic health record systems. While our study’s focus on Gauteng Province and limited sample size requires cautious interpretation, it underscores the need for multi-centre studies with diverse populations to assess the effectiveness of targeted financial support programs. Addressing these challenges requires collaborative efforts between government, healthcare providers and community organisations, with a focus on financial assistance programs. By taking these steps, South Africa can make tangible progress towards achieving SDG 3 and reducing premature cancer mortality.

## Conflicts of interest

The authors declare that they have no conflicts of interest.

## Informed consent

Participants’ consent was obtained via a consent form.

## Author contributions

Portia N Ramashia: primary author, co-conceived the study, determined the research design, data collection and investigation, performed data analysis and interpreted data and drafted the manuscript. Pauline B Nkosi and Thokozani P Mbonane: supervised and co-conceived the study, participated in its design, contributed to the manuscript’s text and content, including revisions and edits. All authors approve of the manuscript’s content and agree to be held accountable for the work.

## Figures and Tables

**Table 1.** An example of illustrative analysis, indicating the pathway from initial quotes to themes.

Initial quote	Initial code(s)	Sub-theme	Theme
I had to borrow money from my family just to pay for the taxi to the hospital...	Expensive transport cost, Financial strain due to treatment	Unaffordable transport costs	Treatment access challenges
The treatment itself is free, but I can’t afford the travel costs.	Heavy treatment cost	Financial barriers to access	Treatment access challenges
I waited three months for my first appointment	Long treatment waiting period	Long waiting times	Delays in diagnosis and starting radiotherapy

**Table 2.** Participant demographic information.

Participant ID	Age	Gender	Cancer diagnosis	Education level	Employment	Distance to facility^1^ (km)
C PT 1	35	Female	Head & neck	Degree	Teacher	37
C PT 2	56	Female	Breast cancer	Degree	Unemployed	51
C PT 3	46	Female	Cervical cancer	Some high school	Driver	25
C PT 4	57	Female	Head & neck	Matriculated	Unemployed	32
C PT 5	55	Female	Cervical cancer	Some high school	Domestic worker	19
C PT 6	68	Female	Breast cancer	Matriculated	Pensioner	14.2
C PT 7	43	Female	Cervical cancer	Some high school	Domestic worker	14
C PT 8	50	Female	Cervical cancer	Some high school	Cleaner	45
C PT 9	72	Female	Cervical cancer	Some high school	Pensioner	45
C PT 10	51	Male	Head & neck	Degree	Teacher	17.4
C PT 11	69	Male	Prostate cancer	Matriculated	Pensioner	55
C PT 12	45	Female	Cervical cancer	Some high school	General worker	16.4
C PT 13	53	Female	Cervical cancer	Some high school	Domestic worker	4
S PT 1	54	Female	Cervical cancer	Some high school	Self-employed (selling clothes)	16
S PT 2	75	Female	Cervical cancer	Primary school	Pensioner	46
S PT 3	54	Female	Cervical cancer	Some high school	Domestic worker	65
S PT 4	19	Male	Head & neck	Some high school	Unemployed	55
S PT 5	43	Female	Cervical cancer	Matriculated	General work (Road maintenance)	16
S PT 6	26	Female	Cervical cancer	Some high school	Unemployed	41
S PT 7	46	Female	Cervical cancer	Diploma	Clerk	41
S PT 8	68	Male	Prostate cancer	Matriculated	Pensioner	39
S PT 9	33	Female	Cervical cancer	Matriculated	Retail	41
S PT 10	58	Female	Cervical cancer	Primary school	Self-employed (selling vegetable)	114
S PT 11	42	Female	Breast cancer	Degree	Teacher	42
S PT 12	50	Female	Cervical cancer	Diploma	Lab assistant	41

**Participant ID**: A unique identifier for each participant, **Age**: The participant’s age at the time of the study, **Gender**: The participant’s gender; **Cancer Diagnosis**: The type of cancer the participant has been diagnosed with; **Education Level**: The highest level of education the participant has completed; **Distance to Facility (km)**: The approximate distance in kilometers between the participant’s residence and the radiotherapy facility [1] Distance was calculated using Google Maps from the participant’s reported address to the main entrance of the radiotherapy facility

**Table 3.** Challenges in accessing radiotherapy services in Gauteng Province, South Africa, Mapped to the 5 A’s Framework.

5A dimension	Challenge	Recommendations	SDG 3 link
Availability	Long wait times: average exceeding 8 months for a first consultation and 13 months to CT simulation.	Prioritise investment in infrastructure and staffing to reduce wait times.	Directly addresses SDG 3.4 by improving timely access to cancer treatment.
Accessibility	Scarcity of facilities resulting in patients traveling long distances and incurring substantial financial burdens	Streamline referral pathways, provide transportation assistance, and establish satellite radiotherapy centres in underserved areas.	Reduces geographical barriers, contributing to SDG 3.8 on universal health coverage.
Affordability	Patients struggling to afford transportation, out-of-pocket expenses, and time off work; Lower socioeconomic status can significantly impact access	Implement targeted financial support programs for vulnerable patients, including subsidies for transportation and accommodation.	Alleviates financial burdens, promoting equitable access to healthcare under SDG 3.8.
Acceptability	Cultural beliefs and stigma surrounding cancer lead to delays in seeking medical care; Normalisation of cancer symptoms, leading to delayed help-seeking	Conduct culturally sensitive awareness campaigns to promote early detection and address misconceptions about cancer treatment. Foster collaboration between conventional medical practitioners and traditional healers.	Improves health-seeking behaviour and reduces stigma, supporting SDG 3.4 and 3.8.
Accommodation	Complex and often inefficient referral processes; echoing the patient-reported difficulties in accessing timely and appropriate radiotherapy services	Streamline referral processes, provide clear communication about treatment processes, and offer patient-centred scheduling.	Enhances the patient experience and ensures that services are responsive to their needs, supporting SDG 3.8.

## Appendix A Interview guide: radiation oncology patients on socio-economic and demographic experiences

1. Introduction and purpose
  - Introduction:
    - Introduce yourself, your affiliation, and your role in the research. Provide a brief overview of the research project: “A Framework to Improve Access to Radiotherapy Services in Gauteng Province, SA.”
  - Interview objective:
    - Explain that the aim of the interview is to understand the socio-economic and demographic experiences of patients in accessing radiotherapy services to help develop a framework for improving access.
2. Demographic information
  - Personal background:
    - Could you provide some basic demographic information (e.g., age, gender, marital status and household size)?
  - Residential information:
    - Where do you live, and how would you describe your community (e.g., urban, rural)?
    - How does your residential location affect your access to healthcare services?
3. Socio-economic factors
  - Financial impact:
    - What financial challenges have you encountered in accessing radiotherapy services (e.g., treatment costs, travel expenses)?
    - Have there been any significant out-of-pocket expenses related to your treatment?
  - Employment status:
    - How has your employment status or job situation influenced your ability to attend treatments or manage related costs?
    - Have you had to make adjustments to your work schedule or job responsibilities due to treatment?
4. Access to resources and support
  - Healthcare resources:
    - What kind of financial assistance or support services have been available to you from the healthcare system or community organizations?
    - Were you informed about any resources or programs that could assist with the costs or logistics of treatment?
  - Community and family support:
    - How has your family or community support system impacted your ability to access and manage your treatment?
    - Are there any additional forms of support or resources you wish had been available to you?
5. Treatment experience
  - Service access:
    - How did you first learn about and access radiotherapy services?
    - Did you experience any barriers or difficulties in scheduling or receiving treatment?
  - Treatment challenges:
    - What challenges have you faced during your treatment related to socio-economic or demographic factors?
    - How have these challenges impacted your treatment experience and adherence?
6. Impact on daily life
  - Quality of life:
    - How have socio-economic and demographic factors affected your quality of life during treatment?
    - What impact have these factors had on your overall well-being and daily activities?
  - Managing challenges:
    - How have you managed the challenges related to socio-economic and demographic factors while undergoing treatment?
7. Recommendations for improvement
  - Improvement Areas:
    - What changes or improvements would you suggest to help overcome socio-economic and demographic barriers to accessing radiotherapy services?
    - Are there specific services or supports that you believe should be added or enhanced?
8. Conclusion
  - Final thoughts:
    - Is there anything else you would like to share about your experience or suggestions for improving access to radiotherapy services?
  - Follow-up:
    - Can we contact you if we have additional questions or need further clarification?
